# Biphasic Memory Impairment and Recovery After Sevoflurane Exposure Are Associated With Time‐Dependent Hippocampal α5‐GABAAR Remodeling

**DOI:** 10.1002/cns.70957

**Published:** 2026-05-27

**Authors:** Sixuan Wang, Lu Chen, Shengran Wang, Chen Zhang, Mengxue Zhang, Zhun Wang, Jinpeng Dong, Qiangwei Liu, Zhonglan Dong, Xiaokun Wang, Ying Dong, Yuan Luo, Yongan Wang, Yiqing Yin

**Affiliations:** ^1^ Department of Anesthesiology, Tianjin Medical University Cancer Institute & Hospital National Clinical Research Center for Cancer Tianjin China; ^2^ Tianjin's Clinical Research Center for Cancer Tianjin China; ^3^ State Key Laboratory of National Security Specially Needed Medicines Academy of Military Medical Sciences Beijing China

**Keywords:** α5‐GABAAR, gephyrin, perioperative neurocognitive disorder, sevoflurane

## Abstract

**Background:**

Dynamic modulation of α5‐GABAAR expression and synaptic distribution plays a pivotal role in neuronal homeostatic plasticity, critically influencing memory processes. This study aims to investigate the spatiotemporal dynamics of α5‐GABAAR in hippocampal subregions (CA1, CA3, and DG) and their behavioral correlation in mice following sevoflurane exposure across eight timepoints.

**Methods:**

Eight‐week‐old female C57BL/6 mice were exposed to 3% sevoflurane for 1 h and they were subjected to trace fear conditioning followed by sevoflurane. Hippocampal tissues were harvested for proteomic analysis and immunofluorescence staining to quantify the expression of α5‐GABAAR and P‐gephyrin. Three‐dimensional spatial colocalization of α5‐GABAAR and gephyrin was reconstructed in IMARIS software.

**Results:**

By integrating trace fear conditioning with molecular profiling, we identified 2 days postexposure (Sev2d) as the critical phase for sevoflurane‐induced memory impairment and 6 days postexposure (Sev6d) as the recovery phase. The time‐dependent biphasic pattern of α5‐GABAAR regulation was demonstrated by proteomics, immunofluorescence, and 3D imaging: (1) At Sev2d, α5‐GABAAR expression and postsynaptic clustering were significantly elevated, which coincided with peak cognitive deficits; (2) by Sev6d, both receptor density and synaptic localization normalized to baseline level, paralleling memory restoration.

**Conclusions:**

These findings indicate that changes in the expression and distribution of α5‐GABAAR are correlated with sevoflurane‐induced memory impairment and recovery, providing potential insights into sevoflurane‐induced memory fluctuation.

## Graphical abstract

Our study reveals that the reversible changes in memory impairment and recovery following sevoflurane with dynamic changes of expression and distribution of α5‐GABAAR. Memory impairment is associated with the upregulation of α5‐GABAAR expression and postsynaptic distribution on day 2 after sevoflurane exposure. Over time, mice can recover memory function by day 6 after exposure with the redistribution of postsynaptic α5‐GABAAR.

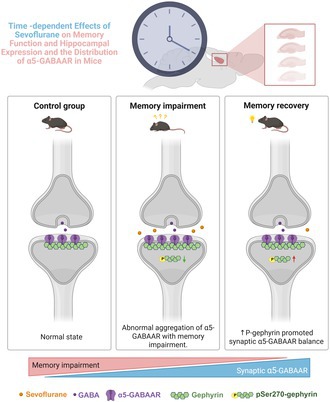

## 1 Introduction

With the continuous increase in surgical volume, the need to prevent and manage anesthesia‐related neurological complications has become increasingly urgent [1]. In 2018, an international consensus established perioperative neurocognitive disorder (PND) as a unified diagnostic term, replacing heterogeneous concepts such as postoperative cognitive dysfunction (POCD). This diagnostic framework classifies PND into four subtypes on the basis of temporal progression [2]. PND is a serious postoperative neurologic complication characterized by memory impairment. The overall incidence of PND ranges from 1.9% to 54% [3, 4, 5, 6, 7, 8, 9, 10]. According to prior clinical studies, this heterogeneity predominantly stems from four confounding factors: (1) variability in surgical types and anesthesia protocols [11, 12, 13, 14, 15, 16, 17, 18]; (2) diversity in age demographics of study populations [13, 15, 17, 19, 20]; (3) methodological biases in cognitive assessment tools [6, 13, 21, 22, 23]; and (4) inconsistency in observation time windows [17, 20, 23, 24, 25, 26]. Notably, the lack of standardized postoperative cognitive evaluation timepoints was identified as the primary confounding factor. These clinical dilemmas highlight the need for studies tracking memory changes at multiple timepoints after anesthesia intervention remain to be addressed.

Sevoflurane, the most widely used inhaled anesthetic in clinical practice, is preferred for diverse surgical procedures because of its low blood‐gas partition coefficient and rapid induction properties. However, clinical studies have indicated that compared with other anesthetics, exposure to sevoflurane is significantly associated with an increased risk of PND [27, 28, 29, 30]. The pathway through which sevoflurane functions as one of the key risk factors for the induction of PND remains uncertain, but studies have shown that the α5‐GABAAR provides an important interaction site for sevoflurane [31, 32, 33]. Although α5‐GABAAR accounts for only approximately 5% of the total GABAAR in the brain [34], its expression accounts for 20%–25% of that in the hippocampal region [35], with dense localization in the CA1/CA3 subfield [36], suggesting its potential involvement in memory regulation.

The involvement of α5‐GABAAR in learning and memory processes has been confirmed in previous studies [37, 38]. Animal models have demonstrated that enhanced α5‐GABAAR activity typically impairs cognition, whereas pharmacological or genetic suppression of α5‐GABAAR function enhances spatial memory performance [39, 40, 41]. Importantly, *Gabra5* knockout mice exhibit significant resistance to the cognitive impairment induced by volatile anesthetics, supporting the necessity of α5‐GABAAR in postanesthesia memory dysfunction [39]. Furthermore, the synaptic localization of GABAAR is tightly regulated by the scaffolding protein gephyrin, whose phosphorylation critically modulates the postsynaptic clustering of GABAARs [42]. It has been shown that phosphorylation at the Ser270 site negatively regulates the number of gephyrin clusters, affecting GABAAR aggregation [43, 44, 45, 46, 47]. Therefore, we hypothesize that changes in α5‐GABAAR expression and distribution after the exposure of sevoflurane correlate with memory function; these changes are closely associated with gephyrin.

In summary, eight critical timepoints were selected on the basis of the key timepoints defined in the latest PND criteria and accounting for the relative maturation rates of mice and humans [48], our study explores and tracks the trajectory of sevoflurane‐induced time‐specific memory and α5‐GABAAR fluctuations in the hippocampus of mice at different cognitive observation timepoints after sevoflurane exposure (0 h, 4 h, 16 h, 1 day, 2 days, 4 days, 6 days, 8 days).

## 2 Methods

### 2.1 Animals and Grouping

Eight‐week‐old female C57BL/6 mice were purchased from SPF (Beijing) Biotechnology Co. Ltd. All the mice were housed in a spf‐grade animal facility and climate‐controlled room on a 12‐h light/dark cycle (lights on from 8:00 a.m. to 8:00 p.m.), with no more than five mice per cage and bedding changed regularly with water and standard laboratory food freely available. All the experimental procedures were conducted in accordance with the Guide for the Care and Use of Laboratory Animals published by the National Institutes of Health. The entire animal study was reviewed and approved by the Animal Ethics Committee of Tianjin Medical University Cancer Institute and Hospital.

The mice were randomly assigned to the control group (Con) or sevoflurane group (Sev). Behavioral tests were performed involving 10 mice in each of the two groups. Three mice in each group were sacrificed for brain tissue preparation.

### 2.2 Trace Fear Conditioning (TFC)

**FIGURE 1:**
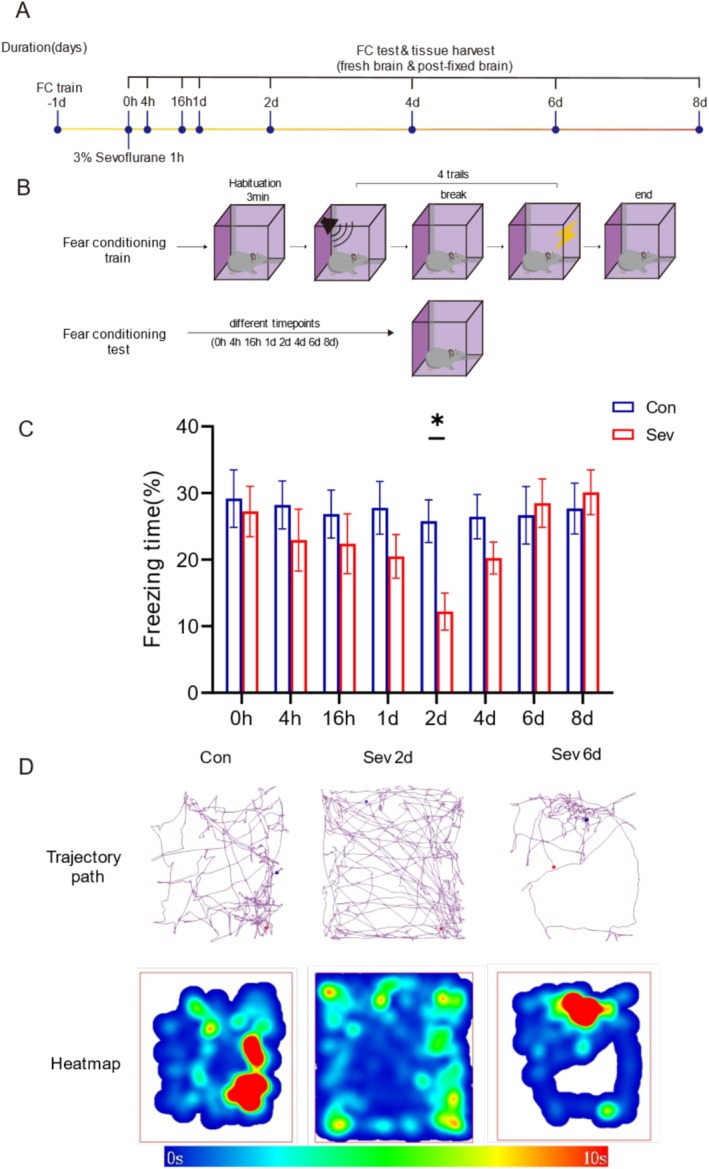
Experimental design and behavioral test results. (A) Timeline of the experiment and mouse grouping. (B) TFC experimental process. (C) TFC results at eight timepoints after sevoflurane exposure (*n* = 10). (D) TFC representative trace and heatmap at key timepoints (2 days, 6 days). The data are presented as the mean ± SEM. Con, control group; Sev, sevoflurane group; Sev2d, 2 days after sevoflurane exposure; Sev6d, 6 days after sevoflurane exposure. Statistical analyses included two‐way and one‐way ANOVA, followed by Šídák's test and Tukey's test. *p < 0.05.

As previously discussed, TFC is strongly associated with α5‐GABAAR‐related memory [49]. TFC was employed to investigate the effect of sevoflurane on contextual memory. All procedures were performed during the photoperiod. The experiments included training, intervention with oxygen or sevoflurane, and testing (Figure 1B).

The day before sevoflurane exposure, two groups of mice were subjected to TFC training. The equipment was cleaned by wiping with 75% ethanol before each experiment, and the mice were placed in a shock box and acclimatized to the environment for 3 min. Then, the mice were given a 3 kHz, 80 db tone stimulus for 30 s with a 3 s interval and a sustained foot shock of 0.8 mA for 2 s following a rest interval of 1 min. The above tone‐electric stimulus was repeated for 4 cycles to strengthen the contextual memory of the mice. Afterward, the mice were returned to their original cages.

Anesthesia was induced on day 0, and TFC tests were performed at eight timepoints after sevoflurane exposure. In the absence of any stimulus, the mice were placed in the same shock box for 8 min, and contextual freezing behavior was recorded. Freezing was defined as the absence of any visible movement except for respiration. The percentage of freezing time was automatically identified and recorded via VisuTrack video analysis software.

### 2.3 Anesthesia

The mice in Sev were sent to an induction chamber made of transparent acrylic acid and ventilated with a mixture of 30% O_2_ for 10 min. Then, the mice were rapidly exposed to 3% sevoflurane (Shanghai Hengrui Pharmaceutical Co. Ltd., China) until the righting reflex disappeared and maintained with 3% sevoflurane and 2 L/min 30% O_2_ for 1 h. The control group of mice inhaled only 30% O_2_ in the induction chamber for 1 h without any additional treatment.

Throughout anesthesia, we used a heat blanket to maintain the core temperature of the mice at 37°C ± 0.5°C. We continuously monitored the vital signs of the mice and the concentration of sevoflurane and carbon dioxide in the induction chamber. After anesthesia, the mice were transferred to a warm resuscitation box and returned to their respective cages where they were allowed to regain consciousness and move freely. All procedures were performed to ensure the comfort of the experimental animals during anesthesia.

### 2.4 Sample Preparation

The sample sizes for each experiment are arranged as follows: Behavioral experiments: *n* = 10 per group; Proteomics: *n* = 3 per group; Immunofluorescence: *n* = 3 per group. Samples were harvested after TFC tests at different timepoints after sevoflurane exposure. Fresh hippocampal tissues were isolated for DIA proteomics. Another batch of whole‐brain samples was fixed by tissue fixation (G1101, Servicebio, China) on a shaker at room temperature for 1 day, after which they were dehydrated through the use of 3 sucrose gradients prior to the preparation of frozen brain sections. Each section was 10 μm thick, and the sections were stored at −80°C for later use. Whole brains were used for immunofluorescence staining in frozen sections.

### 2.5 Immunofluorescence Staining

The sections were removed from the −80°C freezer for rewarming, and sections were selected with a 20×/50× microscope. The sections were permeabilized with saponin‐containing immunostaining permeabilization solution (P0095; Beyotime, China) for 10 min at room temperature and blocked with immunostaining blocking solution (P0102; Beyotime, China) for 1 h at room temperature.

The sections were incubated overnight at 4°C with the following primary antibodies: anti‐α5GABAAR (1:200; ab242001; Abcam, England), Ser270 phosphorylation gephyrin (1:200; 147,318; SYSY, Germany), and anti‐gephyrin (1:100; ab177154; Abcam, England). The next day, the sections were rinsed three times with PBS and subsequently incubated with the following secondary antibodies: goat anti‐mouse IgG (1:500; ab150115; Abcam, England), goat anti‐guinea pig IgG (1:500; ab150185; Abcam, England), and goat anti‐rabbit IgG (1:200; AF488; SAB, USA) for 1 h at 37°C. Afterward, the sections were rinsed three times with PBS and then subjected to DAPI (1:1000; C1002; Beyotime, China) staining. Finally, the sections were sealed with anti‐quenching sealer (P0126‐5 mL; Beyotime, China).

### 2.6 Three‐Dimensional Spatial Distribution Analysis of Immunofluorescence

We employed immunofluorescence (IF) staining to track the changes in the expression of α5‐GABAAR and Ser270‐phosphorylated gephyrin. For each group of data (*n* = 3), 3 layers of brain slices from the anterior, middle and posterior regions of the whole brain were taken from each sample for planar fast scanning (Pannoramic SCAN, 3DHISTECH CaseViewer, Hungary). Fiji was applied to match the brain slices with the standard brain atlas in point‐to‐point correspondence, and the brain regions were standardized to match and delineate them. The brain slices were anastomosed 1:1 to the standard brain atlas using Fiji and Imaris v9.9.0. Finally, the Cell and Surface functions of Imaris v9.9.0 were used to measure the mean fluorescence intensity of α5‐GABAAR and P‐gephyrin in the CA1, CA3 and DG regions. The statistical value of each sample was the mean fluorescence intensity of 3 unilateral hippocampus.

Images of α5‐GABAAR and gephyrin colocalization in the hippocampal CA1, CA3 and DG regions at critical timepoints (2 and 6 days) after sevoflurane exposure were captured with a vertical confocal microscope (LSM 980; Carl Zeiss AG; Oberkochen, Germany) using the Airyscan function. For each group of data, *n* = 3, 7–10 layers of brain slices were selected for each sample, in which at least 3 images were taken for each brain slice and multiple 20 × 20 μm fields of view were randomly selected from each image for statistical analysis. The average value of the multilayer brain slice data was taken as the value of each sample. For imaging data, multiple fields of view were acquired from each animal, and the average value per animal was used as a unit of analysis. Evaluate the colocalization correlation between α5‐GABAAR and gephyrin using the Pearson's coefficient in Imaris Measurement and Coloc functions. Perform 3D rendering of surface maps using the Surface and Snapshot functions in Imaris software.

### 2.7 Proteomics Pathway and Functional Enrichment Analyses

Differentially expressed proteins were identified based on |log_2_ Fold change| > 1 and adjusted *p*‐value < 0.05. We performed Gene set variation analysis (GSVA) to assess the correlation between Con, Sev2d, and Sev6d groups.

For biological process and pathway enrichment analyses, Kyoto Encyclopedia of Genes and Genomes (KEGG), Wiki Pathways (WP), and Gene Ontology (GO) analyses were performed with the R clusterProfiler package. Gene set enrichment analysis (GSEA) was performed using GSEA software (version 4.1.0).

### 2.8 Statistical Methods

In this study, the quantitative results were derived from at least 3 samples and 3 independent experiments, and the data are presented as the mean ± standard error of the mean (SEM). The Shapiro–Wilk test was used to assess the normal distribution of the data. When the data conformed to a normal distribution, the unpaired Student's *t*‐test was used to compare the differences between the two groups. We used two‐way ANOVA with the Šídák multiple comparisons test to compare the results between groups. In addition, we used one‐way ANOVA with Tukey's multiple comparisons test to compare the results between control groups. We have reported the results for all key comparisons, including exact *p* values and effect sizes. All the data were statistically analyzed using GraphPad Prism 9.9.0 (GraphPad, San Diego, CA), and the *p* values are expressed as follows: *p < 0.05, **p < 0.01, and ***p < 0.001.

## 3 Results

### 3.1 Time‐Specific Memory Fluctuations in Mice After Sevoflurane Exposure

To observe the effect of sevoflurane on memory in mice, we performed TFC training one day before sevoflurane exposure and selected 8 timepoints after sevoflurane exposure to assess memory function via the TFC test (Figure 1A,B). When the percentage of freezing time was used as a statistical index, there was no significant difference between the control groups (*p* = 0.9990, *F* = 0.08305), indicating that memory function in mice was not affected by the time interval between observation timepoints (Figure 1C,D). Compared with that in Con, the freezing time in Sev decreased over time, decreasing to the lowest point on the 2nd day after sevoflurane exposure (Sev2d vs. Con2d, *p* = 0.0397, *t* = 3.201) and returning to the control level on the 6th day (Sev6d vs. Con6d, *p* > 0.9999, *t* = 0.3226) (Figure 1C,D). These results suggest that sevoflurane exposure induced memory fluctuations over time in mice, with memory impairment induced at Sev2d and that memory function recovered to control level at Sev6d.

### 3.2 DIA Proteomic Analysis of Sevoflurane‐Induced Memory Impairment

To investigate the potential molecular mechanism underlying memory impairment and recovery, we selected samples from the Con, Sev2d, and Sev6d and subjected them to DIA proteomics to further analyze the differences in gene expression in hippocampal tissue among the three groups.

**FIGURE 2:**
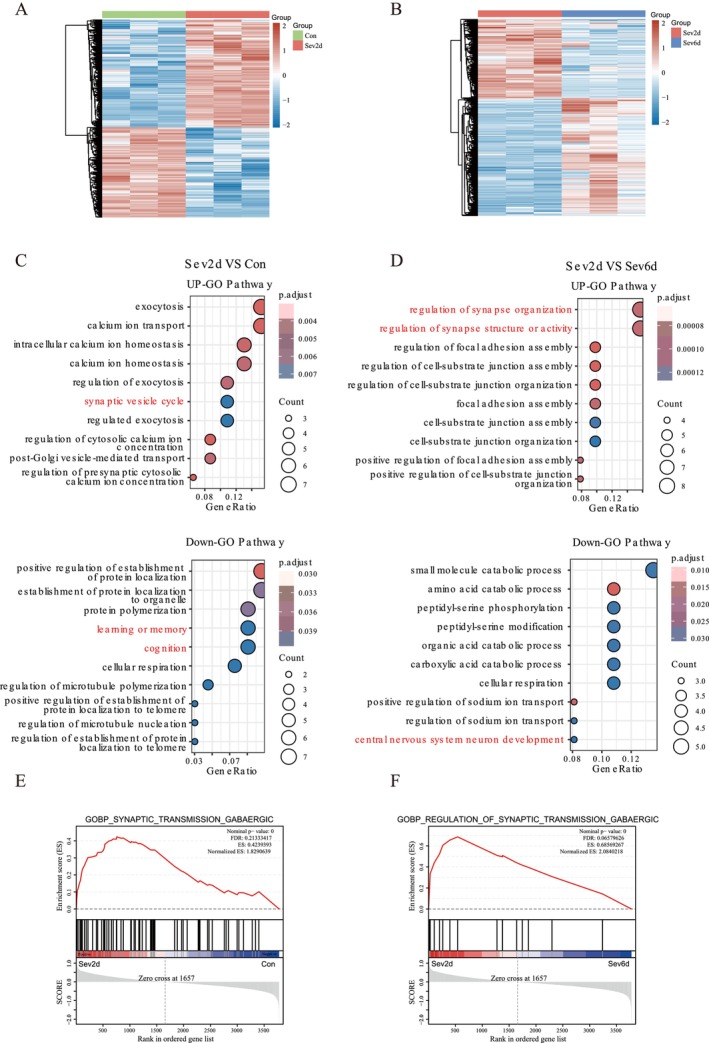
DIA proteomic results for the hippocampus at key timepoints (2 days and 6 days) after sevoflurane exposure. (A, B) Heatmap of genes whose expression was significantly dysregulated between the Sev2d group and the Con and Sev6d groups. Red indicates increased gene expression, and blue indicates decreased gene expression. (C, D) Bubble plots showing upregulated and downregulated enrichment pathways for Sev2d relative to those for Con and Sev6d. The *p* value is adjusted by color; the smaller the *p* value is, the redder the color. The size of the circle indicates the number of genes within the pathway. (E, F) Enrichment analysis using GSEA revealed a trend toward GABAergic changes in the Sev2d group compared with the Con and Sev6d groups. Red indicates a highly enriched pathway. In contrast, blue indicates negative enrichment of pathways.

By comparing Con and memory impairment at Sev2d, by DEGS, we identified a total of 121 differential proteins, of which 51 were upregulated and 70 were downregulated (Figure 2A). By comparing memory recovery at Sev6d and memory impairment at Sev2d, by DEGS, we identified a total of 93 differential proteins, with 53 upregulated and 40 downregulated (Figure 2B). By GO enrichment analysis, compared with Con and Sev6d, Sev2d was shown to be involved in upregulated pathways, such as exocytosis, the synaptic vesicle cycle, and downregulated pathways, such as learning and memory, cognition, and central nervous system neuron development (Figure 2C,D). By GSEA enrichment analysis, Con and Sev2d were compared with Sev6d and Sev2d, and we observed that the GABAergic variation trend was consistent, indicating that the Sev2d GABAergic pathway was enriched and Sev6d GABAergic pathways were restored to the control level.

### 3.3 Sevoflurane Exhibits a Time‐Dependent Effect on α5‐GABAAR Expression

**FIGURE 3:**
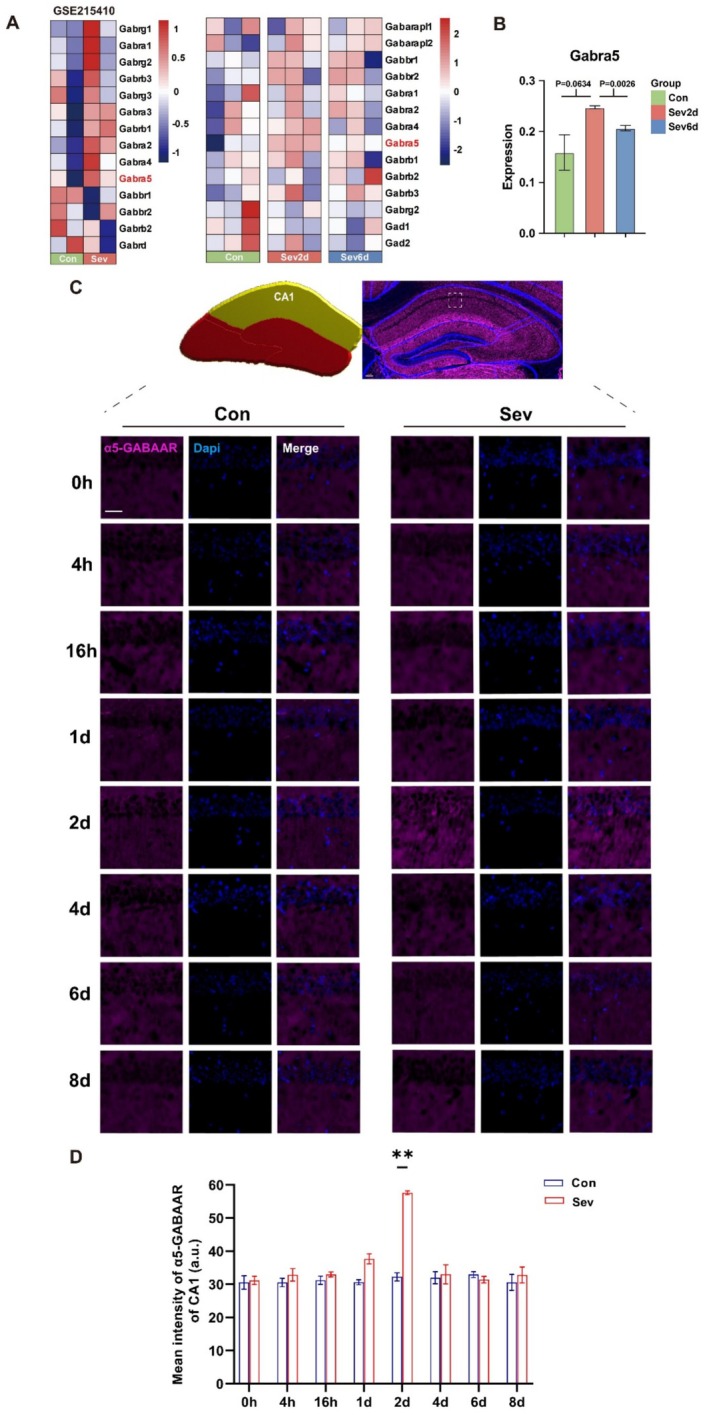
Long‐term axial CA1 α5‐GABAAR expression after sevoflurane exposure. (A) Left panel: Heatmap of the expression of genes related to GABA receptors (GSE215410 dataset). Right panel: GABA receptor‐related gene heatmap comparing Con, Sev2d and Sev6d. Red represents high gene expression, and blue represents negative gene enrichment. (B) Bar graph showing α5‐GABAAR expression in the Con, Sev2d and Sev6d groups. (C) The upper right panel shows a representative brain slice in Imaris software that was matched to a standard brain atlas. The upper left panel shows a 3D model according to the representative brain slice, with the bright yellow region being the statistically significant target brain region CA1. The lower panel shows the tracking result of the long‐term axial α5‐GABAAR (pink) of CA1, as shown in a representative example. (D) α5‐GABAAR expression in the CA1 region at 8 timepoints after sevoflurane exposure (*n* = 3). The data are presented as the mean ± SEM. Con, control group; Sev, sevoflurane group; Sev2d, 2 days after sevoflurane exposure; Sev6d, 6 days after sevoflurane exposure. Scale bar, 1 μm. Statistical analyses included unpaired *t*‐test, two‐way and one‐way ANOVA, followed by Šídák's test and Tukey's test. **p < 0.01.

On the basis of the results of the proteomic analysis, we further explored the expression of GABA‐related receptors in the GABAergic neural pathway. We analyzed the proteomic results of sevoflurane associated with memory impairment (GSE215410 database) to compare the changes in GABAergic‐related genes among the Con, Sev2d, and Sev6d groups. Notably, the trend in the α5‐GABAAR results was consistent, and compared with that in Con, the expression of α5‐GABAAR significantly increased in both groups of mice with sevoflurane‐induced memory impairment. Moreover, the heatmap results show that the expression of α5‐GABAAR of Sev6d was restored to the level of Con (Figure 3A). Analyzing the proteomic α5‐GABAAR expression situation, there was no difference between the Con and Sev6d groups (Sev6d vs. Con, *p* = 0.2444, *t* = 1.364). Sev2d α5‐GABAAR expression was significantly greater than that in both the Con (Sev2d vs. Con, *p* = 0.0634, *t* = 2.548) and Sev6d groups (Sev2d vs. Sev6d, *p* = 0.0026, *t* = 6.702) (Figure 3B).

**FIGURE A1:**
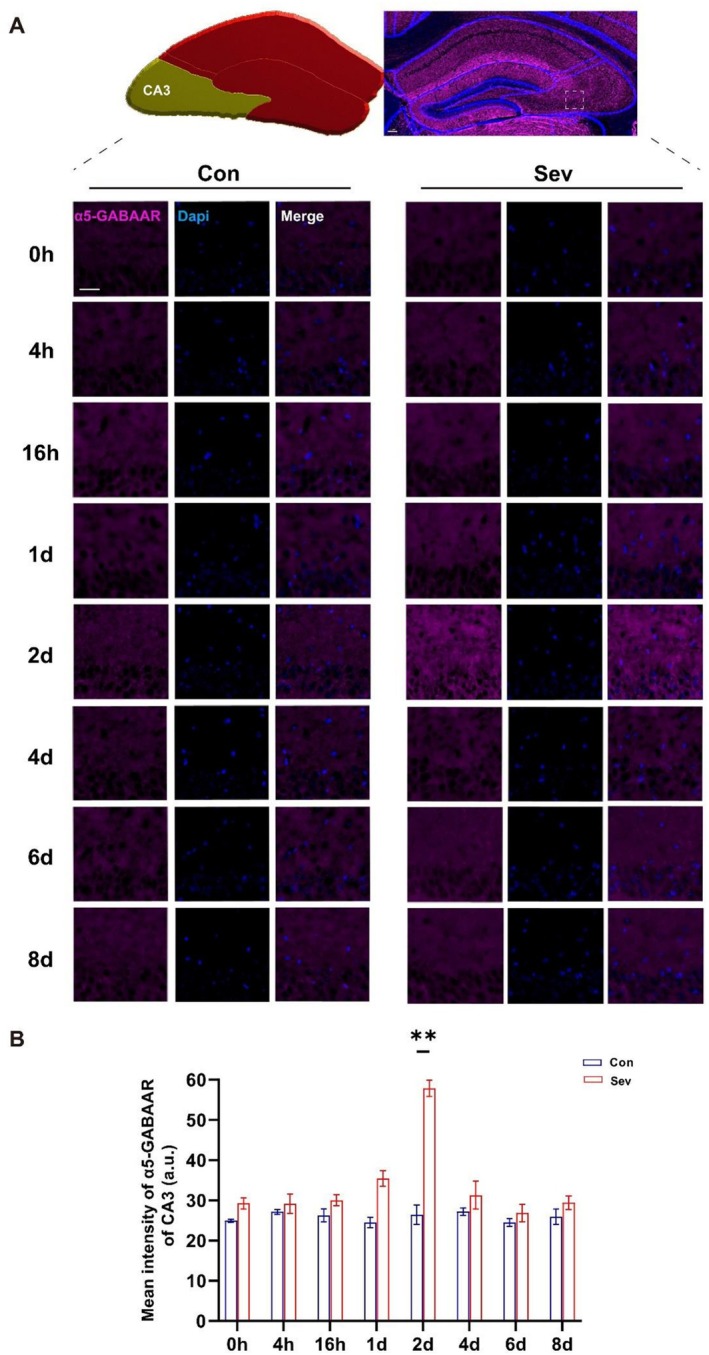
Long‐term axial CA3 α5‐GABAAR expression after sevoflurane exposure. (A) The upper right panel shows a representative brain slice in Imaris software that was matched to a standard brain atlas. The upper left panel shows a 3D model according to the representative brain slice, with the bright yellow region being the statistically significant target brain region CA3. The lower panel shows the tracking result of the long‐term axial α5‐GABAAR (pink) of CA3, as shown in a representative example. (B) Expression of α5‐GABAAR in CA3 at 8 timepoints after sevoflurane exposure (*n* = 3). The data are presented as the mean ± SEM. Con, control group; Sev, sevoflurane group. Scale bar, 1 μm. Statistical analyses included two‐way and one‐way ANOVA, followed by Šídák's test and Tukey's test. **p < 0.01.

**FIGURE A2:**
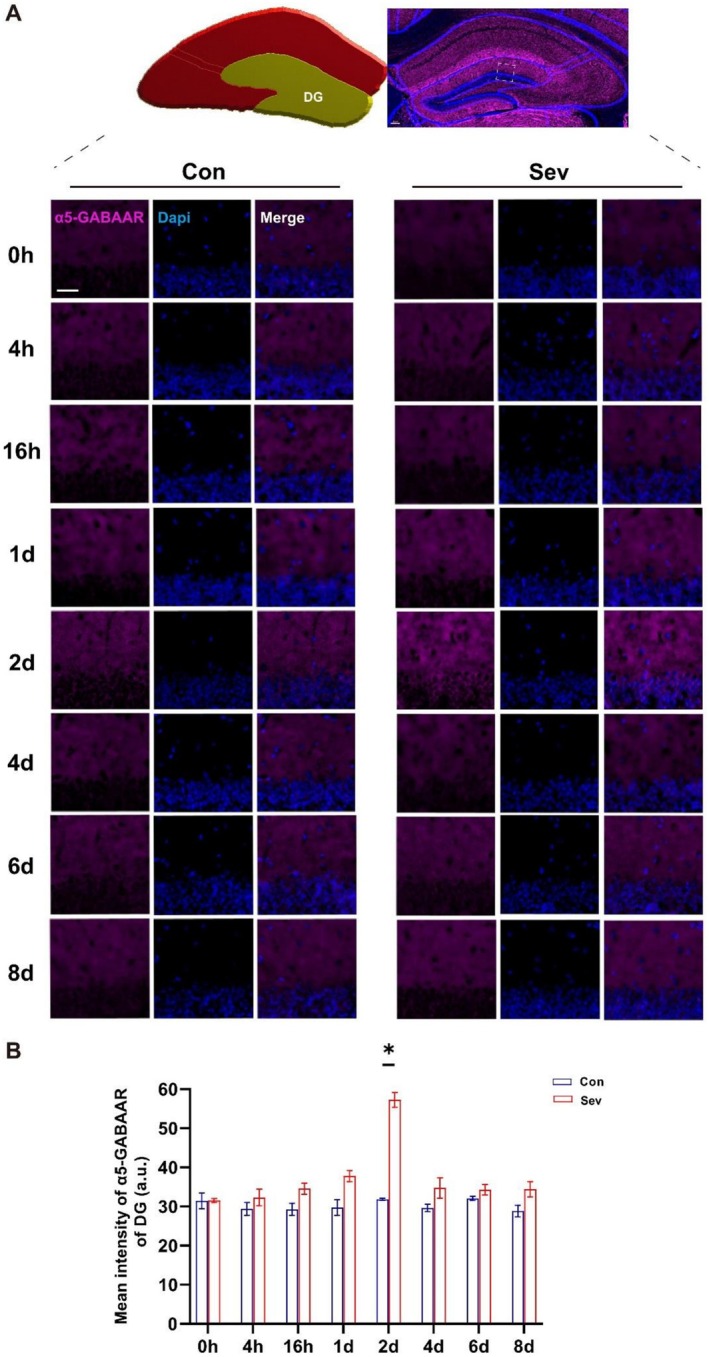
Long‐term axial DG α5‐GABAAR expression after sevoflurane exposure. (A) The upper right panel shows a representative brain slice in Imaris software that was matched to a standard brain atlas. The upper left panel shows a 3D model according to the representative brain slice, with the bright yellow region being the statistical target brain region DG. The lower panel shows the tracking result of the long‐term axial α5‐GABAAR (pink) of the DG, as shown in a representative example. (B) α5‐GABAAR expression in the DG at 8 timepoints after sevoflurane exposure (*n* = 3). The data are presented as the mean ± SEM. Con, control group; Sev, sevoflurane group. Scale bar, 1 μm. Statistical analyses included two‐way and one‐way ANOVA, followed by Šídák's test and Tukey's test. *p < 0.05.

To track long‐term axial α5‐GABAAR expression in the CA1, CA3, and DG regions after sevoflurane exposure, we performed immunofluorescence staining and anastomosis mapping on brain sections (Figure 3C and Figures A1A and A2A). Our results revealed no significant difference in α5‐GABAAR expression among the Con in the brain regions (CA1, *p* = 0.9081, *F* = 0.3676; CA3, *p* = 0.7485, *F* = 0.5984; DG, *p* = 0.6109, *F* = 0.7833), suggesting that α5‐GABAAR does not significantly fluctuate over time in the absence of sevoflurane (Figure 3D and Figures A1B and A2B). Compared with those in Con, α5‐GABAAR expression level in the brain regions increased at Sev2d (CA1, Sev2d vs. Con2d, *p* = 0.0041, *t* = 18.82; CA3, Sev2d vs. Con2d, *p* = 0.0053, *t* = 9.923; DG, Sev2d vs. Con2d, *p* = 0.0368, *t* = 13.43), when memory was most significantly impaired after sevoflurane exposure but then recovered to control level at Sev6d (CA1, Sev6d vs. Con6d, *p* = 0.9478, *t* = 1.167; CA3, Sev6d vs. Con6d, *p* = 0.9821, *t* = 1.004; DG, Sev6d vs. Con6d, *p* = 0.8596, *t* = 1.615) (Figure 3D and Figures A1B and A2B). According to the results of the statistical analysis, the α5‐GABAAR was expressed at different levels in the three subregions of the CA1, CA3, and DG, but the overall trend was consistent over time, and the mean fluorescence intensity of the α5‐GABAAR in each brain region increased on Sev2d.

### 3.4 Mapping of P‐Gephyrin Expression Over Time in Sevoflurane‐Exposed Mice

**FIGURE 4:**
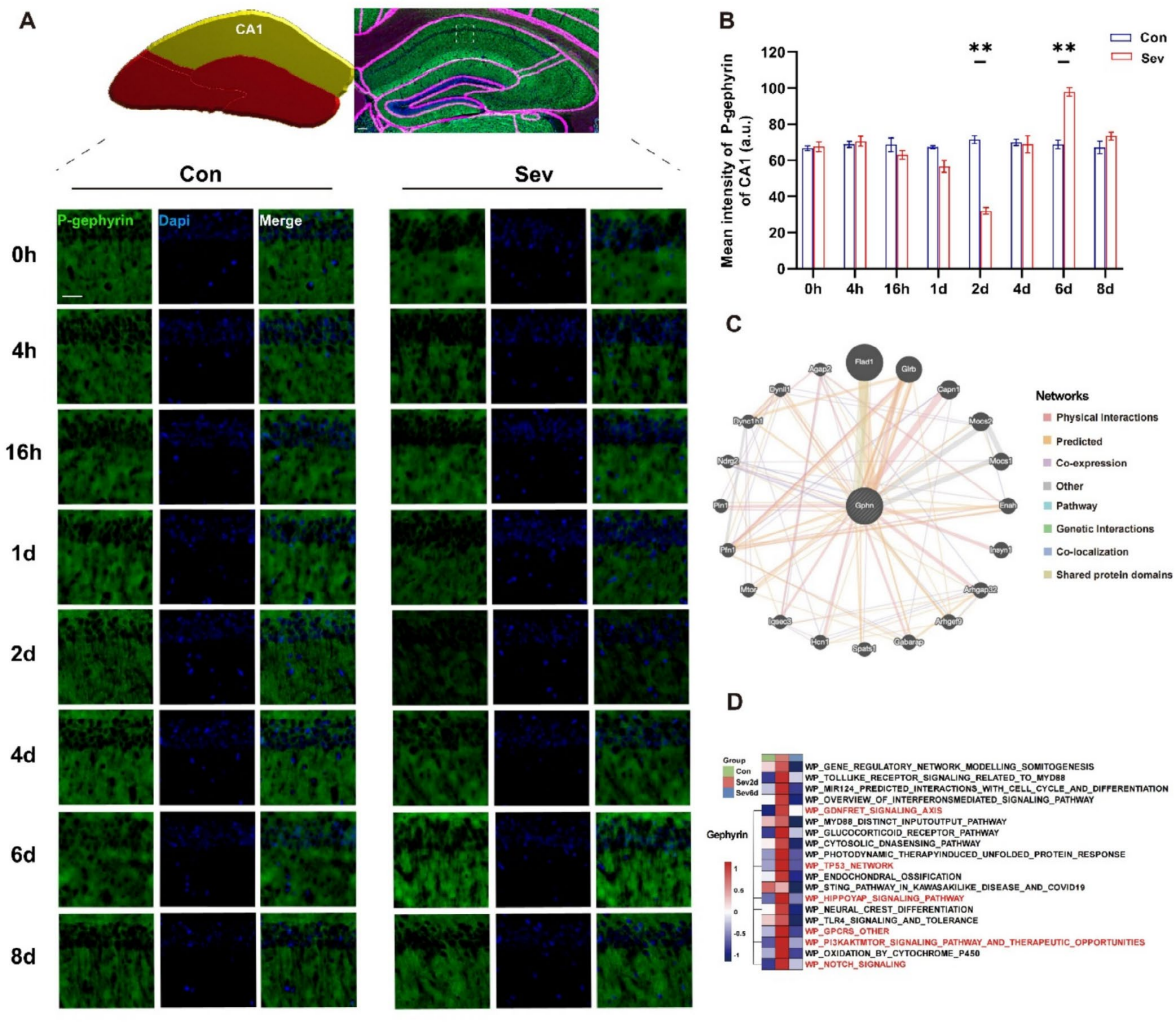
Long‐term axial CA1 P‐gephyrin expression after sevoflurane exposure. (A) The upper right panel shows a representative brain slice in Imaris software that was matched to a standard brain atlas. The upper left panel shows a 3D model according to the representative brain slice, with the bright yellow region being the statistically significant target brain region CA1. The lower panel shows the tracking result of long‐term axial P‐gephyrin (green) of CA1, as shown in a representative example. (B) Expression of P‐gephyrin in the CA1 region at 8 timepoints after sevoflurane exposure (*n* = 3). (C) Screening of differential protein interaction networks that regulate gephyrin. The greater the proportion of circles is, the closer the differential protein is to the gephyrin regulatory mechanism. (D) WP database was used to screen the upregulated genes involved in the regulation of the gephyrin enrichment pathway. The data are presented as the mean ± SEM. Con, control group; Sev, sevoflurane group; Sev2d, 2 days after sevoflurane exposure; Sev6d, 6 days after sevoflurane exposure. Scale bar, 1 μm. Statistical analyses included two‐way and one‐way ANOVA, followed by Šídák's test and Tukey's test. **p < 0.01.

**FIGURE A3:**
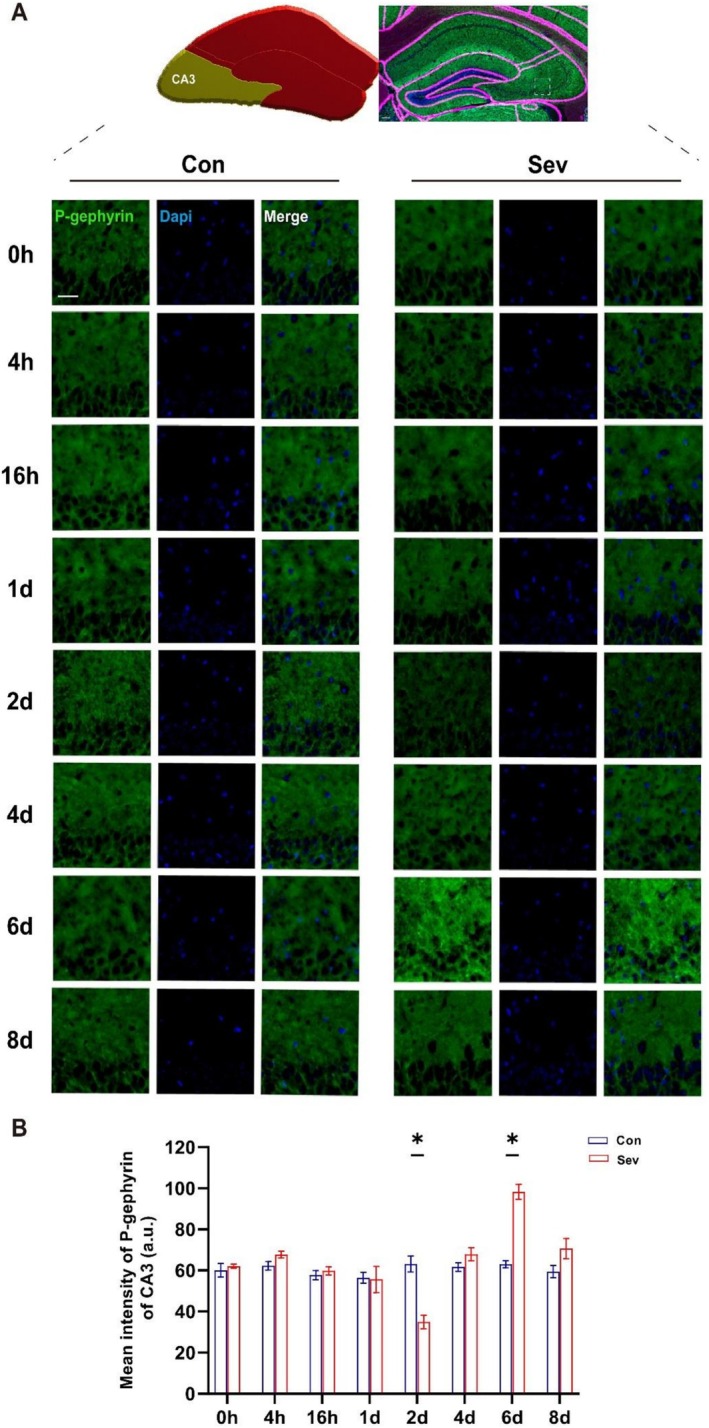
Long‐term axial CA3 P‐gephyrin expression after sevoflurane exposure. (A) The upper right panel shows a representative brain slice in Imaris software that was matched to a standard brain atlas. The upper left panel shows a 3D model according to the representative brain slice, with the bright yellow region being the statistically significant target brain region CA3. The lower panel shows the tracking result of the long‐term axial P‐gephyrin (green) of CA3, as shown in a representative example. (B) Expression of P‐gephyrin in CA3 at 8 timepoints after sevoflurane exposure (*n* = 3). The data are presented as the mean ± SEM. Con, control group; Sev, sevoflurane group. Scale bar, 1 μm. Statistical analyses included two‐way and one‐way ANOVA, followed by Šídák's test and Tukey's test. *p < 0.05.

**FIGURE A4:**
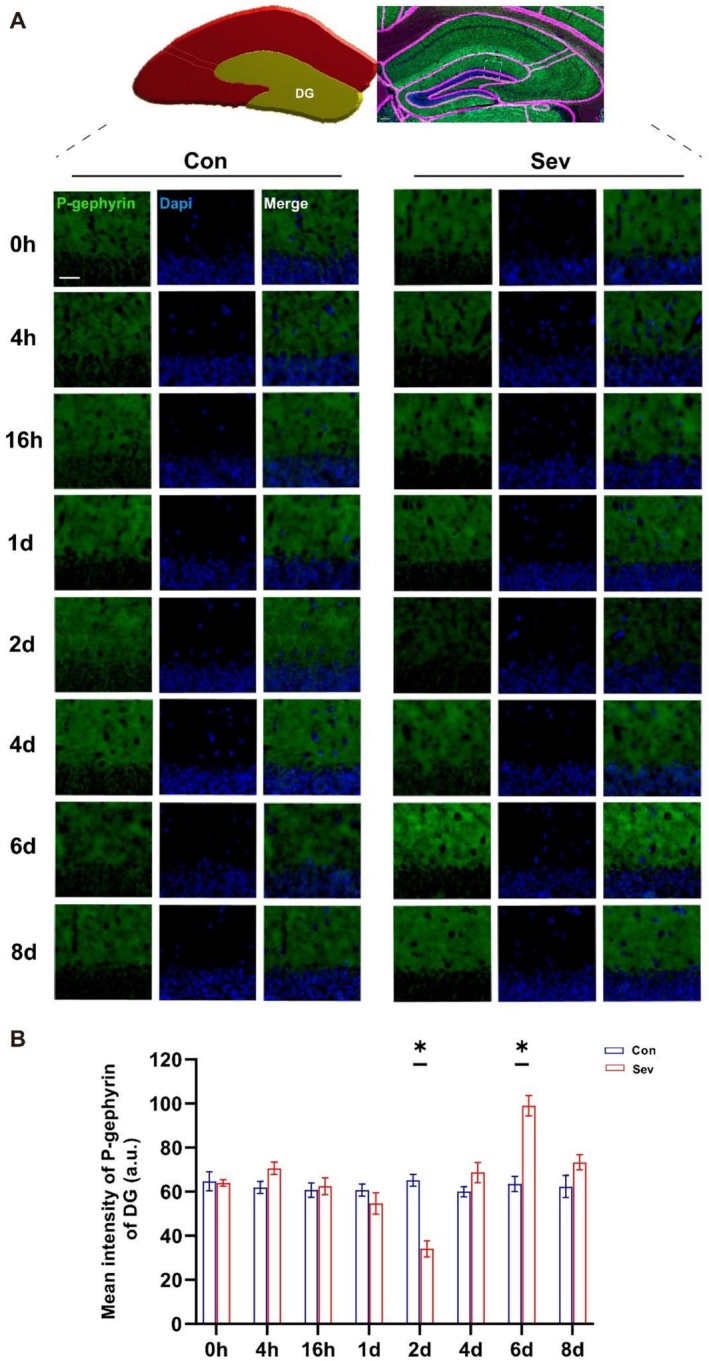
Long‐term axial DG P‐gephyrin expression after sevoflurane exposure. (A) The upper right panel shows a representative brain slice in Imaris software that was matched to a standard brain atlas. The upper left panel shows a 3D model according to the representative brain slice, with the bright yellow region being the statistical target brain region DG. The lower panel shows the tracking result of long‐term axial P‐gephyrin (green) of the DG, as shown in a representative example. (B) Expression of P‐gephyrin in the DG at 8 timepoints after sevoflurane exposure (*n* = 3). The data are presented as the mean ± SEM. Con, control group; Sev, sevoflurane group. Scale bar, 1 μm. Statistical analyses included two‐way and one‐way ANOVA, followed by Šídák's test and Tukey's test. *p < 0.05.

Given that the expression of gephyrin is a key factor influencing the postsynaptic expression and distribution of α5‐GABAAR [50, 51]. Furthermore, gephyrin phosphorylation at the Ser270 site negatively regulates the number of gephyrin clusters in the postsynaptic area [47, 52, 53, 54]. Thus, we measured and plotted long‐term axis P‐gephyrin expression in the CA1, CA3, and DG regions after sevoflurane exposure (Figure 4A and Figures A3A and A4A).

Our results revealed that the expression of P‐gephyrin in Con was not affected by time (CA1, *p* = 0.8598, *F* = 0.4443, CA3, *p* = 0.5637, *F* = 0.8501, DG, *p* = 0.9373, *F* = 0.3133) (Figure 4B and Figures A3B and A4B). In the brain regions, P‐gephyrin expression decreased on Sev2d (CA1, Sev2d vs. Con2d, *p* = 0.0013, *t* = 14.07, CA3, Sev2d vs. Con2d, *p* = 0.0439, *t* = 5.543, DG, Sev2d vs. Con2d, *p* = 0.0258, *t* = 6.854) but increased on Sev6d compared with that in Con (CA1, Sev6d vs. Con6d, *p* = 0.0073, *t* = 8.810, CA3, Sev6d vs. Con6d, *p* = 0.0293, *t* = 8.701, DG, Sev6d vs. Con6d, *p* = 0.0346, *t* = 6.175), with no difference at the other timepoints (Figure 4B and Figures A3B and A4B). Moreover, we screened synaptic activity‐related proteins interacting with gephyrin, including Mtor, Pin1, Pfn1, Ndrg2, Dync1h1, Dynll1, Agap2, Flad1, Glrb, Capn1, Mocs2, Mocs1, Enah, Insyn1, Arhgap32, Arhgef9, Gabarap, Spats1, Hcn1, and Iqsec3, among others (Figure 4C).

In addition, we screened the enriched pathways related to gephyrin among the upregulated pathways, including the PI3K/AKT/MTOR, GDNF/RET, TP53, HIPPOYAP, NOTCH, and GPCR pathways in the Sev2d group by pathway enrichment analysis (Figure 4D).

### 3.5 Colocalization of α5‐GABAAR and Gephyrin After Sevoflurane Exposure Is Changed

Both α5‐GABAAR expression and distribution influence synaptic plasticity, which affects memory [55, 56]. Therefore, we further explored the changes in α5‐GABAAR distribution on sevoflurane‐induced memory by observing the co‐localization between α5‐GABAAR and gephyrin.

**FIGURE 5:**
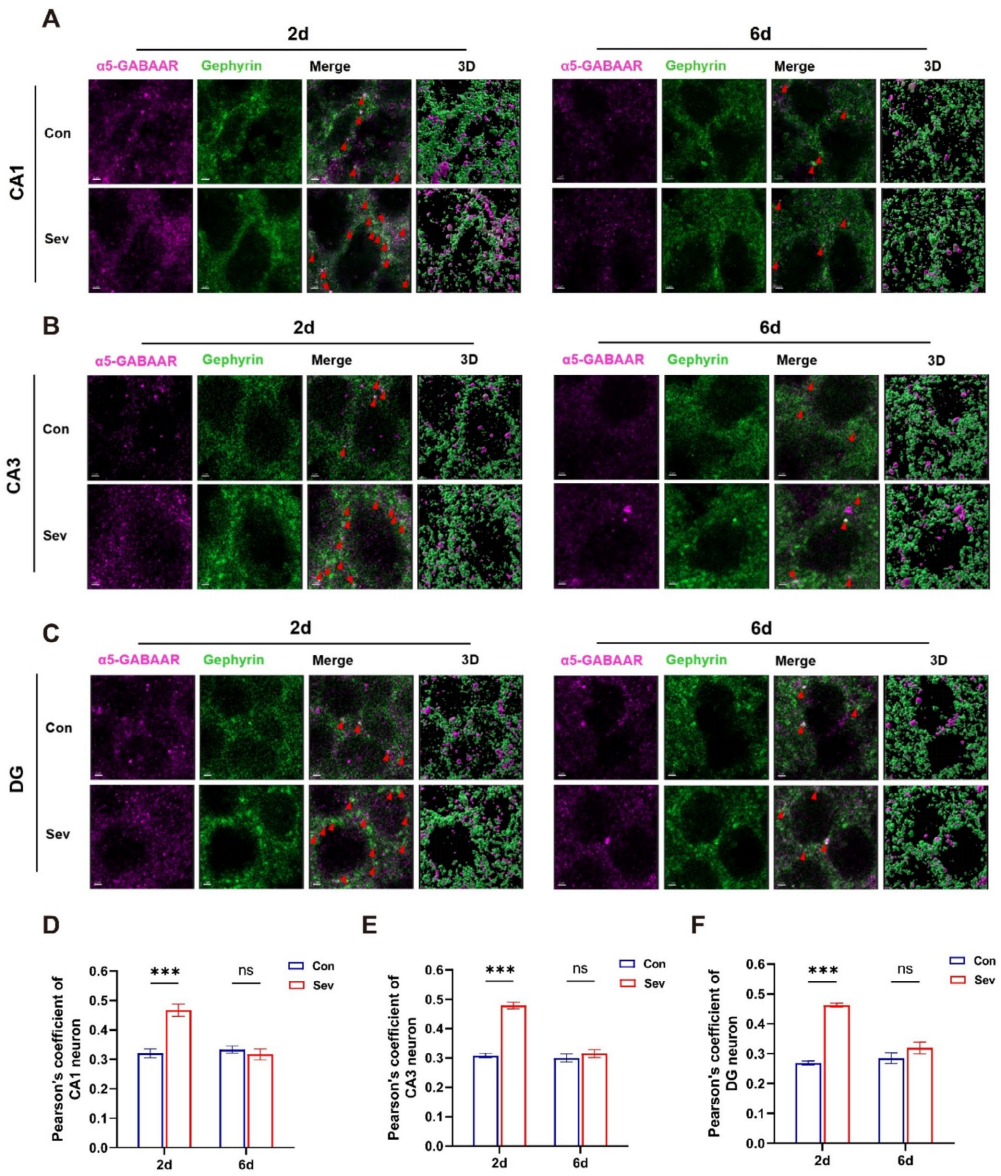
Colocalization of α5‐GABAAR with gephyrin 2 and 6 days after sevoflurane exposure. (A–C) Representative examples of α5‐GABAAR (pink) and gephyrin (green) in the CA1, CA3, and DG regions 2 and 6 days after sevoflurane exposure are shown. (D–F) Colocalization of α5‐GABAAR and gephyrin in the CA1, CA3, and DG 2 and 6 days after sevoflurane exposure; Pearson's coefficient was used as a statistical indicator (*n* = 3). The data are presented as the mean ± SEM. Con, control group; Sev, sevoflurane group. Red arrow: Regions where gephyrin and α5‐GABAAR colocalize. Scale bar, 2 μm. Statistical analyses included two‐way, followed by Šídák's test. ***p < 0.001.

As shown in the figures, the colocalization of α5‐GABAAR with gephyrin was imaged in 3D stereo (Figure 5A–C). The Pearson's coefficient increased in the CA1 (Sev2d vs. Con2d, *p* < 0.001, *t* = 6.102), CA3 (Sev2d vs. Con2d, *p* < 0.001, *t* = 10.31), and DG (Sev2d vs. Con2d, *p* < 0.001, *t* = 9.669) regions when memory impairment occurred on Sev2d compared with that in Con, suggesting that the postsynaptic anchoring of α5‐GABAAR with gephyrin increased (Figure 5D–F).

Immunofluorescence staining (Figure 5A–C) revealed that there was no significant difference in the Pearson's coefficient of the brain regions when memory function was restored on Sev6d compared with that in Con (CA1, Sev6d vs. Con6d, *p* = 0.76, *t* = 0.6916; CA3, Sev6d vs. Con6d, *p* = 0.62, *t* = 0.9275; DG, Sev6d vs. Con6d, *p* = 0.24, *t* = 1.708), suggesting that the postsynaptic distribution of α5‐GABAAR and gephyrin was restored to control level and remained uniform with memory function recovery (Figure 5D–F).

## 4 Discussion

We investigated the reversibility of sevoflurane‐induced memory impairment over time in this study. Temporally, we compared the memory impairment observed at an early timepoint (day 2) after sevoflurane exposure with the recovery of memory function back to baseline level at a later timepoint (day 6). Spatially, we found that the expression and distribution of synaptic α5‐GABAAR were increased in the hippocampal CA1, CA3, and DG subregions on day 2 after sevoflurane exposure and returned to control level by day 6. Together, our findings reveal a spatiotemporal correlation between α5‐GABAAR and sevoflurane‐induced memory fluctuations.

On the basis of the behaviorally identified peak impairment phase (day 2) and recovery phase (day 6), our study used data‐independent acquisition (DIA) proteomics to elucidate time‐dependent changes in hippocampal protein expression. Consistent with the current understanding of the neurotoxicity of sevoflurane, pathways related to cognition, learning and memory, and central nervous system neurodevelopment were significantly downregulated in the Sev2d group compared with those in both the Con or Sev6d groups [57]. These results suggest that the DIA proteomics results corroborate memory impairment at Sev2d. Conversely, pathways associated with exocytosis, synaptic vesicle cycling, the regulation of synaptic organization, and the modulation of synaptic structure or activity were upregulated in the Sev2d group compared with those in the Con or Sev6d groups. These proteomic results are consistent with existing evidence indicating the broad involvement of synaptic activity in sevoflurane‐induced memory impairment. Previous studies have demonstrated that sevoflurane exposure in offspring mice can lead to aberrant dendritic and synaptic development as well as cognitive dysfunction [58]. Similarly, sevoflurane anesthesia in pregnant mice has been shown to induce neurotoxicity in fetal and offspring mice, manifested as reduced expression of synaptic markers and impaired learning and memory [59].

On the basis of our proteomic analysis, α5‐GABAAR was specifically identified as potentially involved in the temporal process of memory impairment and recovery. Immunofluorescence analysis revealed that the expression of α5‐GABAAR in various hippocampal subregions (CA1, CA3 and DG) exhibited significant temporal characteristics following sevoflurane exposure: it peaked on day 2 postexposure and returned to baseline level by day 6. In addition, DIA proteomics data also revealed an increase in α5‐GABAAR expression at 2 days and recovery at 6 days. Studies have confirmed that specific inhibitors of α5‐GABAAR can reverse cognitive impairment or improve memory function [60, 61, 62]. In addition, Gabra5 knockout mice do not exhibit short‐term memory deficits after isoflurane exposure, and Gabra5^−/−^ mice demonstrate superior spatial learning and memory performance [39, 41], confirming the central role of this receptor subtype in anesthesia‐related cognitive dysfunction. Our study has found that following sevoflurane exposure, the time‐dependent change of α5‐GABAAR expression correlates with memory fluctuations. The alteration in the expression of α5‐GABAAR and the temporal consistency of memory fluctuations further indicate that α5‐GABAAR may play a significant role in the memory impairment caused by sevoflurane.

In addition to being influenced by α5‐GABAAR expression level, homeostatic synaptic plasticity is also associated with its distribution [43, 63]. The synaptic localization of α5‐GABAAR is regulated by its anchoring protein, gephyrin, which is a major scaffolding protein at inhibitory postsynaptic sites [44, 64]. Immunofluorescence analysis revealed that on Sev2d, the phosphorylation level at the Ser270 site of gephyrin was significantly reduced in the CA1, CA3, and DG subregions. We observed increased postsynaptic distribution of α5‐GABAAR in the Sev2d compared with Con. As the observation period progressed to the recovery phase (day 6), the contextual memory of the mice returned to the level of Con. The phosphorylation level of gephyrin at the Ser270 site increased, and we simultaneously observed that the postsynaptic distribution of α5‐GABAAR returned to baseline. Consistent with our findings, Tyagarajan et al. demonstrated that the phosphorylation of the gephyrin at Ser270 negatively regulates the number of gephyrin clusters, thereby influencing GABAAR aggregation [45]. Correspondingly, we observed that the temporal changes brought by phosphorylated gephyrin were consistent with the changes in the distribution of α5‐GABAAR. This suggests that sevoflurane‐induced changes in α5‐GABAAR distribution may be regulated by gephyrin. Our dynamic findings reveal that sevoflurane exposure not only regulates receptor expression level but also modifies the phosphorylation status of gephyrin.

Through differential gene screening and systematic literature mining, we identified several GABAergic pathway‐related proteins in our proteomics dataset, including Nrxn2, PKCγ and CDK5. Specifically, nrxn2 is known to be functionally associated with GABAAR expression and clustering [65]. CDK5 positively modulates GABAAR clustering, in part through phosphorylation of the scaffolding protein gephyrin [66]. In general, sevoflurane anesthesia induces a coordinated multi‐protein alteration that shifts the balance of α5‐GABAAR trafficking. The concurrent downregulation of PKCγ [67], Pdpk1 [68], Ywhaz [69], and Trim3 [70] likely impairs receptor internalization and recycling, allowing α5‐GABAARs to accumulate at synaptic sites without proper turnover. However, these molecules are only preliminary results obtained from our proteomic analysis. Their regulatory effects on the distribution and expression of α5‐GABAAR, as well as their roles in modulating cognitive function, still require further experimental validation.

We acknowledge several limitations regarding the generalizability of our findings. First, the present study used young female C57BL/6 mice. However, several studies have adopted young female mice to investigate anesthetic‐induced cognitive impairment [71, 72]. In the meanwhile, some sex‐specific studies have demonstrated that female individuals are more susceptible to anesthetic‐induced cognitive impairment [73]. Second, although the sample size for immunofluorescence assays was relatively limited, statistically significant differences were clearly detected even with the current sample cohort, demonstrating that the observed alterations in receptor expression are robust and consistent. Future studies should incorporate animals of different ages and sexes with an increased sample size to validate whether the receptor expression and distribution mechanisms identified in this work are generalizable to clinically relevant populations.

## 5 Conclusion

In conclusion, cognitive impairment can occur on the second day after sevoflurane exposure, and this impairment is reversible, with cognitive function returning to normal on the sixth day following sevoflurane exposure. Meanwhile, the expression and distribution of α5‐GABAAR, as well as the phosphorylation level of gephyrin, exhibit dynamic changes consistent with cognitive function. These results suggest that sevoflurane‐induced cognitive impairment may be related to alterations in gephyrin, as well as changes in the expression and distribution of α5‐GABAAR.

## Author Contributions

Yiqing Yin and Yongan Wang designed the experiments. Sixuan Wang and Lu Chen wrote the manuscript. Sixuan Wang, Shengran Wang, Qiangwei Liu, and Mengxue Zhang performed the experiments. Zhonglan Dong, Xiaokun Wang, Ying Dong, and Chen Zhang analyzed data. Sixuan Wang, Zhun Wang, Jinpeng Dong, and Yuan Luo edited the images and revised the manuscript. All the authors contributed to the article and approved the submitted version.

## Funding

This work was supported by The Science & Technology Development Fund of Tianjin Education Commission for Higher Education (2022ZD063).

## Ethics Statement

This research involved animal subjects and it complies with ARRIVE guidelines. The entire animal study was approved by the Ethics Committee for Animal Research of Tianjin Medical University Cancer Institute and Hospital, with ethics approval number No. 2024026.

## Conflicts of Interest

The authors declare no conflicts of interest.

## Data Availability

All the original data or deidentified data that support the findings of this study are available from the corresponding author upon reasonable request.
